# Spatial organization of organelles in fungi: Insights from mathematical modelling

**DOI:** 10.1016/j.fgb.2017.03.006

**Published:** 2017-06

**Authors:** Congping Lin, Gero Steinberg

**Affiliations:** a Mathematics, University of Exeter, North Park Road, Exeter EX4 4QF, UK; b School of Biosciences, University of Exeter, Stocker Road, Exeter EX4 4QD, UK; c Donders Chair, University of Utrecht, Department of Biology, Padualaan 8, 3584 CH Utrecht, The Netherlands

**Keywords:** EE, early endosome, PO, peroxisome, MT, microtubule, ODE, ordinary differential equation, Organelle motility, Mathematical modelling, Spatial organization, Active transport

## Abstract

Mathematical modelling in cellular systems aims to describe biological processes in a quantitative manner. Most accurate modelling is based on robust experimental data. Here we review recent progress in the theoretical description of motor behaviour, early endosome motility, ribosome distribution and peroxisome transport in the fungal model system *Ustilago maydis* and illustrate the power of modelling in our quest to understand molecular details and cellular roles of membrane trafficking in filamentous fungi.

## Highlights

• Modelling of dynein motility reveals a stochastic role in dynein comet formation.
• Modelling helps to elucidate mechanisms in spatial organization of early endosomes.
• A combination of diffusion and directed motion distributes ribosomes and peroxisomes.

## 1 Introduction

**Fig. 1:**
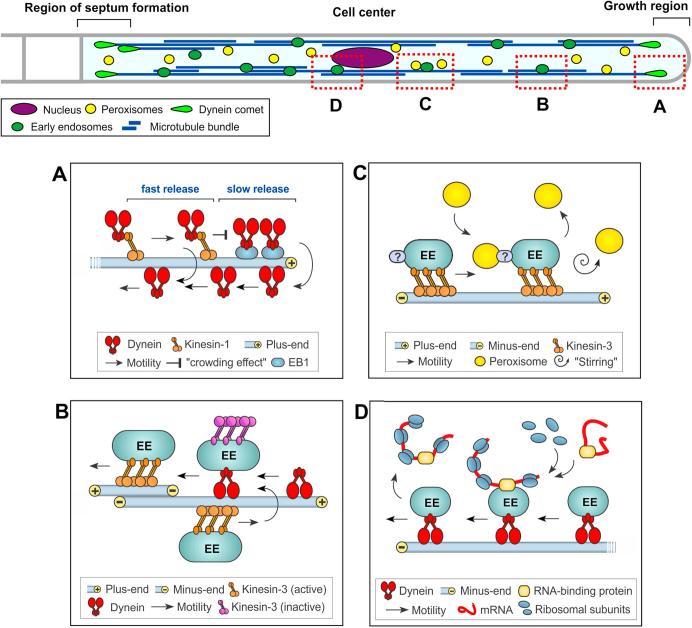
Aspects of intracellular motility that have been investigated using mathematical modelling. Top panel shows a schematic drawing of a hyphal cell of *U. maydis*. The cell grows are one pole (“Growth region”), while septa are continuously formed at the other end (“Region of septum formation”). Microtubule bundles run along the length of the hyphal cell. They are utilized by the molecular motors kinesin-3 and dynein, which transport organelles (such as early endosomes and peroxisomes) in a bi-directional fashion (Steinberg, 2011, Steinberg, 2016). Mathematical modelling helps to elucidate the mechanism and cellular role of these trafficking (red dotted boxes A–D): A: Kinesin-1 delivers dynein to the polar plus-ends of MTs, where dynein accumulates in a “dynein comet”. This comet consists of ∼55 dynein motors and is formed by an active, EB1-based retention and a “crowding effect” of stochastic motion of motors at MT plus-ends identified from mathematical modelling. Observation of native dynein motors reveals that two dynein populations in the comet are released at different rates. Further details can be found in Schuster et al. (2011a). B: The MT array consists of uni-polar regions near the cell ends and anti-polar bundles near the center. 3–4 kinesin-3 motors take early endosomes to the microtubule plus-ends. During this delivery, dynein motors, released from the “comet” can bind to the organelles. This appears to inactivate the kinesin-3 motors, which can take over again within the region of anti-polar bundling. This change to kinesin-3-based transport requires the release from dynein. Such a mechanism is also supported by mathematical modelling. Further details can be found in Schuster et al., 2011b, Schuster et al., 2011c. C: Early endosomes constantly move in a bi-directional fashion (only plus-directed moving early endosomes are shown), thereby generating turbulences (“stirring”) in the cytoplasm which enhance the diffusion (so called as “active diffusion”) of organelles, such as peroxisomes and lipoid droplets (only peroxisomes are shown). In addition, both organelles can transiently bind to moving early endosomes via unknown linker proteins (“?”). Modelling reveals that the combination of active diffusion and dragging of peroxisomes ensures mixing and even distribution of the organelles in the fungal cell. Further details can be found in Lin et al., 2016, Guimaraes et al., 2015. D: Transient binding to early endosomes is also required to distribute entire polysomes. These structures are constantly formed at the nucleolus. After export from the central nucleus into the cytoplasm, transient binding to moving endosomes (only minus-directed moving early endosomes are shown) via the RNA-binding protein Rrm4 (Becht et al., 2006) ensures their distribution in the cell. Mathematical modelling reveals that this “hitchhiking” mechanism is required for even distribution of the protein translation machinery. Further details can be found in Higuchi et al. (2014).

Mathematical modelling provides a powerful way to understand the fundamental principles of biological processes (Tomlin and Axelrod, 2007). In particular, intracellular transport, driven by molecular motors that move their “cargo” along the cytoskeleton, has been described by stochastic models (overview in Bressloff and Newby, 2013). Modelling has provided valuable insights into how motors coordinate their activity to maintain bi-directional transport (Müller et al., 2008). A major challenge for a mathematical model is the availability of robust quantitative experimental data. This includes accurate description of motor numbers on organelles, transport velocities and the characteristics of the cytoskeletal organization. Indeed, such data must be obtained from living cells. This maximizes the accuracy of the model and, thereby, the chance that the theoretical description provides a “realistic” scenario in the living cell. Filamentous fungi are well suited to provide such data for three reasons: (1) they are relatively simple model systems that provide an accessible amount of information without too much background noise (e.g. few organelles that move), (2) there are available construction of mutants in the system (e.g. Schuster et al., 2011a), (3) they are genetically tractable and this, for example, allow visualisation of native protein levels by fusing endogenous proteins to fluorescent tags (e.g. Schuster et al., 2011b, Lin et al., 2016). This can be combined with sophisticated live cell imaging to provide quantitative information on protein numbers. In addition, the tip-growing fungal hypha itself provides an interesting architecture, with apical extension being supported by local exocytosis and long-range transport (Steinberg, 2007). In fact, modelling of tip growth provided valuable insights into the principles of hyphal and mycelial extension (e.g. Bartnicki-Garcia et al., 1989, Davidson et al., 2011, Fuhr et al., 2011, Fuhr et al., 2012). In this review, we focus on modelling of various aspects of intracellular transport in filamentous fungi (Fig. 1 top panel).

## 2 Modelling dynein accumulation at microtubule plus-ends

Molecular motors are key players in intracellular transport in filamentous fungi (Steinberg, 2011, Egan et al., 2012). Long-range motility along microtubules (MTs) is mediated by (1) the motor complex dynein/dynactin, which moves towards MT minus-ends, and (2) kinesins, which deliver their cargo to MT plus-ends. In filamentous fungi, kinesin-1 recycles the dynein/dynactin motor complex back to MT plus-ends (Zhang et al., 2003, Lenz et al., 2006). Here, dynein accumulates in a “comet-like” structure (Fig.1A), where it acts as a “loading zone” for cargo binding and retrograde transport in *Ustilago maydis* and *Aspergillus nidulans* (Lenz et al., 2006, Zhang et al., 2010). To gain insight into the mechanism by which the dynein comet is formed, we have develop a mathematical model (Ashwin et al., 2010, Lin et al., 2011, Schuster et al., 2011a). The model considers anterograde and retrograde dynein moving along a single MT and change in direction of movement (=turning), and includes a 2% loss of arriving dynein, due to a release from the microtubule into the cytoplasm. The loss of dynein reflects the observed escape rate for early endosomes (EEs) at plus ends and assumes a similar fidelity between the kinesin-3 delivering EEs and kinesin-1 delivering dynein. Based on experimentally determined rates of turning, transport frequencies of dynein entering the apical region and velocities of motors, modelling of the stochastic motion of dynein motors, suggests that ∼25 dynein motors accumulate at a microtubule plus end (Schuster et al., 2011a). Video 1 shows such stochastic motion and dynamic maintenance of ∼25 dynein in steady state of dynein trafficking near MT plus end from a simulation of the model. However, quantitative live cell imaging in *U. maydis* shows that the dynein “comet” consists of ∼55 motors (Schuster et al., 2011a, Steinberg, 2014). Interestingly, these motors fall into 2 populations; (1) about 60% of the motors show a slow turn-over rate (T_(1/2)_: ∼98 s). The other 40% are more dynamic (T_(1/2)_: ∼10 s), suggesting a different retention mechanism. Thus, modelling and experimental data independently infer the existence of two populations of motors: (1) one concentrating at the microtubule plus end due to “crowding effect” from stochastic motion of motors, and (2) dynein motors that are retained at the microtubule plus end by protein-protein interaction (Fig.1A). Indeed, this has been confirmed in *U. maydis*, showing a specific interaction of dynactin and EB1-like proteins in the dynein “comet” (Schuster et al., 2011a). This modelling approach has thus provided a new insight into the mechanism of dynein accumulation at microtubule ends and reveals an important role of stochastic motion in organising the dynein comet at the end of MTs.

Video 1Modelling stochastic behaviour of dynein motors at the plus end of a microtubule. The video shows dynein dynamics in a steady state, based on of stochastic behaviour of the motor from the model, at the plus end of a microtubule. Note that all 13 proto-filaments of the MT are shown in one plane. Time in seconds is indicated in the lower right. Further details on the modelling can be found in Schuster et al. (2011a). Note that, in living cells of *U. maydis*, ∼54 dynein motors accumulate at apical microtubule plus ends. It has been reported that only ∼50% of these motors accumulate due to stochastic motion of motors, whereas the other 50% are kept at plus ends via specific protein-protein interactions. The latter is not included in this simulation.
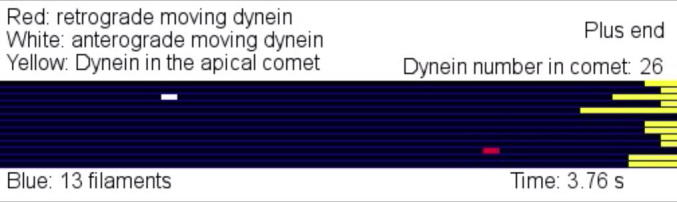

## 3 Modelling bi-directional early endosome motility

In *U. maydis* cells, EEs move rapidly in a bidirectional manner. This motility is driven by kinesin-3 and dynein motors (overview in Steinberg, 2014). While most EE runs are found to be short, some EEs travel over up to 100 µm along a bi-polar MT array, which requires cooperation of both dynein and kinesin-3 (Schuster et al., 2011b). Interestingly, only kinesin-3 is bound constantly to the EEs, whereas dynein interacts transiently (Schuster et al., 2011c), see Fig.1B. This suggests that EEs get loaded onto dynein in transit (“loading on the run”). The interaction of dynein with EEs is mediated by Hok1, a protein with similarities to hook proteins in animal cells. It has been shown in *U. maydis* and *A. nidulans* that these hook proteins are part of a bigger protein complex that mediates binding of dynein to EEs (Bielska et al., 2014, Zhang et al., 2014, Yao et al., 2015). Schuster et al. have developed a theoretical model to describe this phenomenon of “loading on the run” (Schuster et al., 2011c). The model assumes that kinesin-3 delivers EEs from sub-apical regions of the cell to the hyphal tip, whilst dynein is released from the “dynein comet” at microtubule plus-ends after a stochastic pausing time. This modelling approach determines the anterograde and retrograde run length of organelles by the stochastic binding and unbinding of dynein, and suggests that the probability of dynein interaction with EEs underlies the observed bidirectional behaviour of the organelles.

Hyphal cells carry an anti-polar MT array, with uni-polar MTs at both cell poles (Schuster et al., 2011b). To mimic EE transport in an entire cell, Lin et al. considers a lattice model composed of two MTs partially bundled in an anti-polar manner with unipolar MTs at two ends (Lin et al., 2013). The model allows EEs “hopping” from one MT to another, which enables EEs to travel the entire length of the cell. Modelling suggests that a high rate of EE hopping between MTs or a high turning rate at minus end can prevent the formation of unwanted EE clusters, and thus keep EEs moving along MTs as observed in Schuster et al., 2011a, Schuster et al., 2011b. Moreover, this modelling indicates that “hopping” between anti-polar MTs largely affects the relative contribution of kinesin-3 and dynein to EE trafficking. Thus, modelling suggests that EE behaviour at minus ends, in particular its hopping behaviour between MTs, is critical in governing the EE spatial organization.

In addition, *U. maydis* contains long MT bundles, which are composed of a number of MTs (Schuster et al., 2011b). EEs are evenly distributed along this MT array (Schuster et al., 2011b). In contrast to the lattice model in Lin et al. (2013)), Gou et al. ignore the hard-core interaction between EEs, and develop a theoretical model (Gou et al., 2014) based on a system of ordinary differential equations (ODEs), to describe the way in which motors and the bi-polar MT array organize spatially the EE compartment in *U. maydis*. This ODE model takes account of (1) average MT bundle polarity resulting from a number of MTs that are modelled by polymerization and depolymerisation processes with published rates (Steinberg et al., 2001) and the assumption that isotropic MT nucleation was inhibited at cell ends, and (2) four different populations of EEs and transitions among the four populations (Gou et al., 2014, Dauvergne and Edelstein-Keshet, 2015). Among the four populations, two populations are EEs driven by kinesin-3 motors in either left or right going direction (which depends on the MT orientation), and the other two populations are EEs driven by dynein in either left or right going direction. Moreover, the model assumes space-independent constant transition rates among the four populations. Modelling results shows that EE distribution is in agreement with observed EE distribution in wild-type cells, dynein mutants and kinesin-3 mutants shown in Schuster et al. (2011b). Thus, modelling suggests that no space-specific regulation except the regulation for the inhibition of MT nucleation at cell ends is required to achieve even distribution of EEs.

## 4 Modelling peroxisome dynamics

Peroxisomes (POs) are important organelles, involved in oxidative stress protection, in lipid transfer, and in signal transduction (Rodríguez-Serrano et al., 2009, Kohlwein et al., 2013, Schrader et al., 2015). Fungal POs move rapidly along MTs. But surprisingly, their motility is driven by transient interaction with EEs; (Guimaraes et al., 2015, Salogiannis et al., 2016, Steinberg, 2016; Fig. 1C). It has been shown recently that constant EE motility mixes the cytoplasm in *U. maydis* (Lin et al., 2016). This turbulence increases random PO motion. Such energy-dependent increase in random motility is known as “active diffusion” (Almonacid et al., 2015). Together with directed transport along MTs, active diffusion of POs opposes a slow drift of the organelles towards the hyphal cell tip. The apical PO drift is facilitated by tip-ward delivery of myosin-5 “cargos” to the growing hyphal apex (Lin et al., 2016). We have developed a mathematical model to understand the relative importance of the various forces acting on POs (the polar drift, active diffusion and directed transport) for even cellular distribution and interaction of POs (Lin et al., 2016). The model is based on coupled drift-diffusion equations and experimentally derived parameters. It includes experimentally measured or estimated diffusion rates, tip-ward drift velocity, directed transport velocity, transition rates between diffusive and directed PO motility, as well as accounting for changes in direction of motor-driven transport. Modelling results suggest that directed transport, and to a lesser degree, active diffusion both contribute to overcome the polar drift forces. This combination of directed transport and active diffusion ensures even distribution of POs, which is essential for cellular homeostasis (Schrader et al., 2015). Moreover, the model predicts that directed transport and active diffusion contribute to the mobility and mixing of POs, which is of particular importance over short ranges and ensure effective interaction between the organelles. Video 2 shows a simulation for such mixing of POs in a cylinder space from the model, published in Lin et al. (2016), under different conditions. This modelling approach has thus revealed that PO mobility and mixing requires both, active diffusion and directed transport. This mechanism ensures even distribution of POs and frequent interaction, required for proper function of the organelles.

Video 2Mixing of POs from the model. Movie show the mixing of POs in a defined virtual segment of the hyphal cell (10 µm in length and 2 µm in diameter; a 2D-projection of this space is shown). Mixing of two populations of POs is simulated (red POs starting on the right end of the space and blue POs starting at the left end of the space). Our work has recently shown that POs move within the fungal cell by motor-driven motility along the cytoskeleton (directed transport) and by collisions with early endosomes, which enhances their diffusive motion (active diffusion). In this simulation, we test if both mechanisms contribute to PO mixing in the fungal cell. The simulation demonstrates that both, active diffusion and directed transport cooperate to increase the mobility of POs, which results in rapid mixing of POs. More details on the modelling behind this simulation can be found in Lin et al. (2011). Time in seconds is given below.
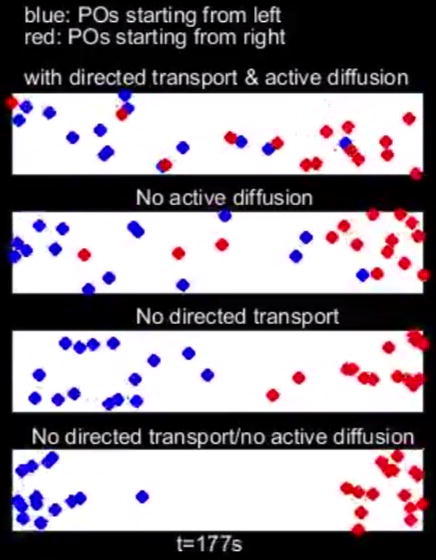

## 5 Modelling ribosome distribution

Ribosomes translate messenger RNA into proteins. They are formed at the nucleolus and subsequently spread throughout the cytoplasm. Live cell imaging studies in *U. maydis* reveals an even distribution of ribosomes in the fungal hypha. Considering that ribosomes are constantly generated at the central nucleus in *U. maydis* hyphal cells, Higuchi et al. find that the diffusion process with the experimentally determined ribosome diffusion coefficient is not sufficient to describe the even distribution of ribosomes (Higuchi et al., 2014). They also find that EEs transiently interact with polysomes, via an RNA-binding protein (Higuchi et al., 2014; Fig. 1D). To further understand how ribosomes are spread evenly in the cell, they have developed a mathematical model, considering both diffusion and directed transport of ribosomes. The model also assumes a constant generation of ribosomes at nucleolus and a reduction of ribosome number at the hyphal tip (Higuchi et al., 2014). This modelling approach suggests that ribosomes undergo passive diffusion, but also are moved within the cell by motor-driven directed transport. Their combined activity results in even distribution of ribosomes. Indeed, live cell imaging has revealed that ribosomes do, indeed, move along microtubules (Higuchi et al., 2014, Baumann et al., 2014). Thus, mathematical modelling reveals that motor-driven directed transport is required to ensure the even distribution of ribosomes.

## 6 Conclusion

Modelling provides a powerful approach towards a deeper understanding of biological processes (Tomlin and Axelrod, 2007). Recent work in the corm smut fungus *U. maydis* uses such theoretical approach to complement and inform live cell imaging experiments. Modelling helps to elucidate the mechanism of dynein accumulation at the plus ends of MTs (Schuster et al., 2011a), bidirectional EE transport (Schuster et al., 2011c, Lin et al., 2013, Gou et al., 2014) and the distribution and mobility of ribosomes and POs in hyphal cells (Higuchi et al., 2014, Lin et al., 2016). In these studies, modelling not only described dynamic processes; it also informs experimental strategies. This iterative process leads to a better understanding of intracellular transport in fungi.
